# Antibody fragments as nanoparticle targeting ligands: a step in the right direction

**DOI:** 10.1039/c6sc02403c

**Published:** 2016-09-16

**Authors:** Daniel A. Richards, Antoine Maruani, Vijay Chudasama

**Affiliations:** a Department of Chemistry , University College London , 20 Gordon Street , London , WC1H 0AJ , UK . Email: daniel.richards.11@ucl.ac.uk ; Email: v.chudasama@ucl.ac.uk ; Tel: +44 (0)207 679 2077

## Abstract

Recent advances in nanomedicine have shown that dramatic improvements in nanoparticle therapeutics and diagnostics can be achieved through the use of disease specific targeting ligands. Although immunoglobulins have successfully been employed for the generation of actively targeted nanoparticles, their use is often hampered by the suboptimal characteristics of the resulting complexes. Emerging data suggest that a switch in focus from full antibodies to antibody derived fragments could help to alleviate these problems and expand the potential of antibody–nanoparticle conjugates as biomedical tools. This review aims to highlight how antibody derived fragments have been utilised to overcome both fundamental and practical issues encountered during the design and application of antibody–targeted nanoparticles.

## Abstract 2

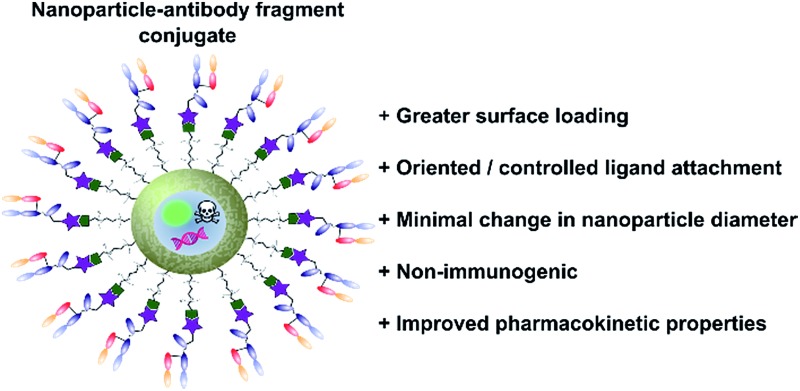
Recent advances in nanomedicine have shown that dramatic improvements in nanoparticle therapeutics and diagnostics can be achieved through the use of disease specific targeting ligands.

## 1. Introduction

The last twenty years have seen a rapid, and accelerating, increase in the use of nanoparticles for biomedical applications. From a conceptual standpoint it is not difficult to understand why; various nanoparticles are now at a stage of being tuneable, functionalisable and biocompatible vehicles that can safely transport large quantities of cargo through the body. This enables the delivery of entities at concentrations significantly higher than traditional methods.^1^ This factor, in combination with the ease in which the surface of nanoparticles can be decorated with high affinity disease-specific targeting ligands to enhance selective delivery, means that they have a plethora of downstream therapeutic and diagnostic applications. A large variety of chemical and biological molecules have been explored for this enhanced targeting purpose, including: novel small molecules, sugars, fatty acids, proteins, peptides, antibodies, and aptamers.^1–7^ Of these, antibody based targeting ligands have become incredibly popular due to their unique *in vivo* properties and high target specificities.^8–11^ Whilst the contributions of other targeting ligands should not be ignored, this review focuses on the use of antibodies, or more specifically their associated fragments, as targeting ligands for nanoparticle-based therapeutic and diagnostic tools. To ensure broad accessibility of the review content, a brief overview of common nanoparticle (Section 2.1) and antibody (Section 2.2) scaffolds used in this context will be given.

## 2. Antibody–decorated nanoparticles

### 2.1 Nanoparticle structure

**Fig. 1:**
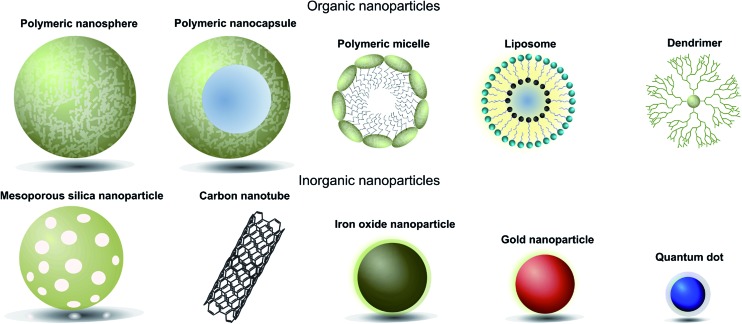
Pictorial representation of different types of nanoparticles used in biomedical applications.

When designing nanoparticle–antibody conjugates for biomedical applications several considerations regarding the structure of the nanoparticle are important. The nanoparticle must be biologically inert, stable under physiological conditions, move freely through the body, securely encapsulate chemical entities (where applicable), and contain a surface which is easily conjugated to the desired targeting antibody. In the case of therapeutics, it is also important to consider the mechanism by which the nanoparticle vehicle will release cargo and whether this will be compatible with other aspects of the overall construct. The most successful approaches strike a delicate balance between the properties of the nanoparticle, the targeting antibody, and where appropriate the encapsulated cargo. Fortunately, a great deal of research has been done on the design and modification of nanoparticles over the last 20 years, providing a rich pool of work from which suitable vehicles can be selected for antibody conjugation. Nanocarriers can be broadly categorised as organic or inorganic,†
†Hybrid organic–inorganic particles will not be focused on in this review. and each of these will be discussed in turn (Fig. 1, Table 1).^4^

**Table 1:** A table summarising the different types of nanoparticles with focus on material used, cargo attachment, and their various advantages & disadvantages

Nanoparticle	Material(s)	Cargo attachment	Advantages	Disadvantages
Liposomes	Self-assembling lipid bilayer	Encapsulated within the hydrophilic core	Easily synthesised, biocompatible, high internal loading	Highly sensitive to structural changes and nature of payload
Polymeric micelles	Hydrophobic polymer core surrounded by hydrophilic polymeric chains	Encapsulated within the hydrophobic core	Small, biocompatible, able to incorporate highly hydrophobic cargo	Highly sensitive to structural changes, poor release profiles
Polymeric nanospheres/nanocapsules	Solid hydrophobic polymer matrix with optional aqueous core (nanocapsule)	Embedded in the polymer matrix or within the core	High loading capacity, flexible loading capabilities, reliable release profiles	Difficult to purify and poor store properties
Dendrimers	Highly branched polymer matrix	Embedded in the polymer branches	Highly soluble, non-immunogenic, high loading capacity, controlled synthesis	Lacking data on toxicity and biocompatibility
Iron oxide nanoparticles	Iron oxide core surrounded by biocompatible coating	Attached to the surface/surface coating	Innate magnetic properties	No internal loading capacity
Gold nanoparticles	Solid gold particles typically coated with PEG chains	Attached to the surface/surface coating	Innate optical and photothermal properties	No internal loading capacity, poor biocompatibility and biodegradability
Mesoporous silica nanoparticles	Mesopores surrounded by a silica framework	Encapsulated within the mesopores	High loading capacity, good biodegradability	Issues with physiological stability, rapid clearance rates
Carbon nanoparticles	Graphite arranged in either a sheet or cylindrical conformation	Attached to the carbon backbone	Innate optical and electrical properties, high surface loading capacities	Poor biodegradability, organ accumulation
Quantum dots	Typically a cadmium selenide core with a zinc selenide cap	Attached to the surface/surface coating	Innate optical properties, high extinction coefficients	No internal loading capacity, potential toxicity issues

#### 2.1.1 Organic nanoparticles

##### Liposomes

Liposomal nanoparticles were first developed near the genesis of nanomedicine and have since become one of the most widely utilised vehicles for encapsulating chemical payloads, with several formulations having gained FDA approval.^12^ They comprise natural lipids with polar and non-polar components which self-assemble into colloidal particles. Whilst early liposomal nanoparticles suffered from issues of stability and rapid clearance, the introduction of surface ligands such as polyethylene glycol (PEG) chains has helped to address these drawbacks.^12,13^ The main advantages of liposomal nanoparticles created from state-of-the-art technologies lie in their excellent biocompatibility, ease of synthesis/functionalisation, and their ability to safely encapsulate a variety of small molecules.^4,6,14^ However, they are limited by a high level of sensitivity to structural change(s) and have demonstrated highly specific cargo-dependency, thus decreasing their universal appeal and broad applicability.^6,14^

##### Polymeric micelles

Polymeric micelles consist of a core of aggregated hydrophobic polymers surrounded by hydrophilic polymeric chains. Their small size and hydrophilic nature allow them to avoid uptake by the reticuloendothelial system, significantly increasing their circulation time.^15^ Their hydrophilic exterior also allows polymeric micelles to effectively and safely encapsulate very hydrophobic drugs for safe transport through the body.^16^ As with liposomal nanoparticles, polymeric micelles also demonstrate excellent biocompatibility.^17^ However, poorly controlled release profiles of encapsulated cargo, and a high sensitivity to structural change(s), mean that there is still significant scope for improvement.^4^

##### Polymeric nanoparticles

Polymeric nanoparticles can be further categorised as either nanospheres or nanocapsules. Nanospheres consist of a solid polymer matrix which is able to encapsulate hydrophobic drugs, whilst nanocapsules contain an aqueous hydrophilic core that is more amenable to the loading of hydrophilic payloads such as DNA/RNA.^10^ This payload flexibility increases the versatility of polymeric nanoparticles, making them attractive candidates as nanocarriers. Additionally, it has been shown that the release rates of encapsulated payloads are constant and proceed on clinically relevant time scales.^6^ Nonetheless, despite these favourable characteristics, polymeric nanoparticles are not simple to purify and do not store well, making them a poor choice for applications that require large scale production.^18^

##### Dendrimers

Dendrimers are branched polymer complexes generated through highly controlled successive polymerisation steps. This leads to a nanoparticle which consists of an initiator core contained within branched polymer chains. These polymer chains are generally synthetic, although examples that employ natural polymers such as sugars and amino acids have been reported.^19^ Their highly regulated synthesis enables excellent control over shape and size – important parameters for medical applications.^20^ They also display excellent solubility and have been shown to be non-immunogenic.^21^ Whilst dendrimers have several excellent qualities, research into their use in the biomedical field is still early stage. Further studies to establish their biocompatibility and toxicity are ongoing and will be pivotal to their further application.

#### 2.1.2 Inorganic nanoparticles

##### Iron oxide nanoparticles

Iron oxide nanoparticles generally consist of an iron oxide (typically Fe_3_O_4_) core surrounded by a dextran coating to improve the physical properties of the complex. The application of these nanoparticles commonly centres on their innate magnetic properties, which allow them to act as excellent MRI contrast agents and tools for therapeutic magnetic hypothermia.^22,23^ This dual functionality has led to superparamagnetic iron oxide nanoparticles (SPIONS) being used as theranostic tools, *i.e.* chemical entities which display both therapeutic and diagnostic properties. However, the lack of a spacious “core” or any porous space leads to low loading volumes,^24^ an issue for many applications. Whilst the generation of hybrid iron oxide/polymer-based nanoparticles has gone some way towards addressing these issues, the current situation is not ideal.^23^

##### Gold nanoparticles

Gold nanoparticles have been extensively studied for use in biomedical applications due to their interesting size dependent physicochemical and optical properties. For example, their ability to produce heat upon absorbance of near-infrared light has been explored for use in photothermal therapy, whilst the ability to enhance optical processes such as absorbance and fluorescence has led to widespread use in the field of biosensors and imaging agents.^25,26^ However, their non-hollow structure precludes internal loading,^4^ and they also tend to suffer from poor biodegradation and questionable biocompatibility.^27–29^

##### Mesoporous silica nanoparticles

Mesoporous silica nanoparticles (MSNs) consist of mesopores (2–50 nm pores) surrounded by a silica framework. These nanoparticles have a high surface area to volume ratio which affords them a large loading capacity. MSNs have also demonstrated good biocompatibility and biodegradability, desirable features for biomedical purposes.^30,31^ However, stability issues and rapid clearance rates significantly restrict the use of MSNs from certain applications.^32–34^

##### Carbon nanoparticles

Carbon nanoparticles, such as carbon nanotubes, comprise a single layer of graphite in either a sheet or cylindrical conformation. Excellent loading capacities, unique optical and electrical properties, and low synthetic costs make them promising candidates for several applications, especially imaging and diagnostics.^35,36^ Unfortunately, issues of poor biodegradability,^37^ pulmonary damage,^29,38^ and undesirable organ accumulation^29,39,40^ have hindered the adoption of carbon based nanoparticles for *in vivo* applications.

##### Quantum dots

Quantum dots (QDs) most commonly consist of a cadmium selenide core with a zinc selenide cap, although many other combinations exist. QDs emit bright colours and also display size dependent optical properties, making them ideal for imaging or biosensing technologies.^41^ Whilst potential toxicity issues have to-date limited their utility *in vivo*, recent advances are helping to overcome these remaining hurdles.^41–43^

### 2.2 Antibody structure and function

**Fig. 2:**
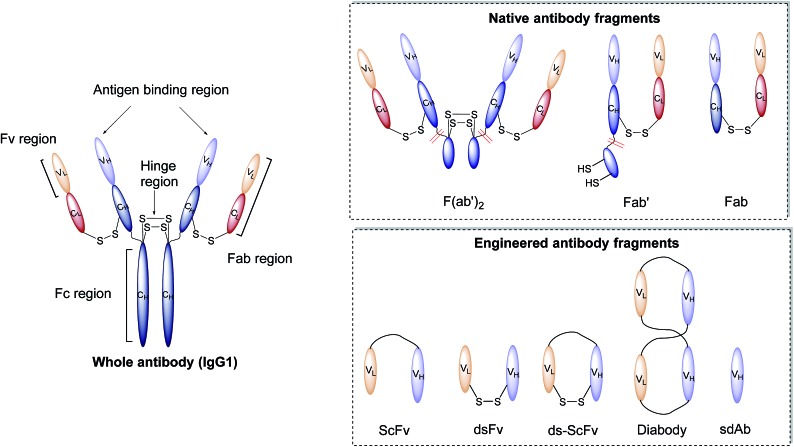
Graphic representations of whole antibody (IgG1) and various fragments.

Antibodies, or immunoglobulins (Ig), are large glycoproteins found in all vertebrate life forms. These essential proteins are involved in several key processes within the immune system including complement dependent cytotoxicity (CDC), opsonisation, phagocytosis, and antibody-dependent cytotoxicity (ADCC). To date, five major classes of immunoglobulin have been discovered, IgA, IgD, IgE, IgG and IgM, each characterised by unique structural characteristics. IgGs represent the dominant class of human immunoglobulins and can be further divided into four sub-types; IgG1, IgG2, IgG3 and IgG4. Although the IgG sub-types show significant sequence variation in key regions, they share a common overall structure. IgG antibodies consist of four protein chains; two identical *ca.* 25 kDa light chains (*i.e.* L-subscript) and two identical *ca.* 50 kDa heavy (*i.e.* H-subscript) chains. These chains contain multiple domains which are characterised by their degree of sequence variability. The N-termini of the chains converge in the variable domain (V) to form the antigen-binding region. Further from the terminus, the structure becomes more conserved, leading to the area being designated the constant region (C). The heavy and light chains are held together by several interchain disulfide bonds and considerable non-covalent interactions to form a Y-shaped structure. The overall structure can be broadly divided into two distinct segments; the fragment antigen-binding (Fab) region and the fragment crystallisable (Fc) section. Fabs can be further divided into variable (Fv, V_H/L_) and constant (C_H/L_) regions (Fig. 2).^44^

#### 2.2.1 Antibody fragments

In addition to being integral to the function of parent immunoglobulins, the individual protein domains of antibodies can be isolated or expressed and have found extended use in biomedical research. Through careful and precise disassembly of a full antibody, researchers have been able to isolate and individually employ the Fab, Fab′, F(ab′)_2_, and Fv regions of antibodies to great effect (Fig. 2).^45^ Additionally, advancements in protein engineering and expression have allowed for the generation of novel classes of antibody fragments such as the ScFv, ds-Fv, ds-ScFv, single domain antibodies (sdAb), and diabodies (Fig. 2).^46–50^ All of these antibody fragments retain at least one antigen-binding region, meaning that the function of active targeting is still present. These individual fragments have then been exploited as part of nanoparticle–antibody fragment conjugates, leading to several interesting studies of the use of these targeting ligands for selective nanoparticle delivery. Given recent advancements in phage display techniques for the generation of antibody derived fragments,^51^ a surge in interest in their use as targeting ligands is unsurprising. Several excellent reviews have been written on the design, production, and applications of antibody fragments, with focus on their merits relative to whole immunoglobulins,^52–57^ and as such this will not be covered in detail in this review.

### 2.3 Nanoparticle–antibody fragment conjugates

**Fig. 3:**
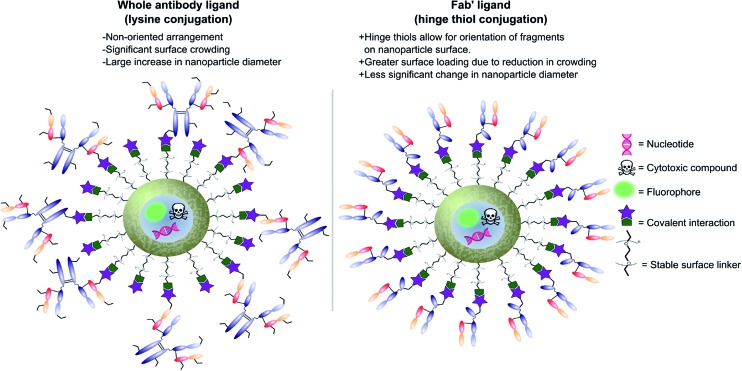
Graphic representations comparing whole antibody and antibody fragment (Fab′) targeting ligands for nanocarriers.

Antibodies function by targeting specific antigens that are expressed only on the surface of diseased cells, or heavily overexpressed on these cells relative to healthy cells. As these antigens are present solely, or majorly, on the surface of the target diseased cells, antibodies can conceptually be exploited to courier nanoparticles (and also their cargo) through the body and enable selective delivery/targeting. Whilst this approach was first conceptualised in the early 1980's, practical and theoretical limitations at the time (*e.g.* insufficient methods for generating and evaluating antibody–decorated nanoparticles) prevented significant progress in the area. Advancements in both antibody expression techniques and nanoparticle design over the past few decades have enabled a more thorough exploration of nanoparticle–antibody conjugates, which has resulted in a rapid expansion of the field. Early developments focused almost entirely on using full antibodies as targeting ligands, primarily due to the wealth of available information on both their generation and modification. However several issues associated with the use of full antibody ligands, such as immunogenicity,^9^ rapid elimination,^58^ poor stability,^59,60^ and lower than expected efficacy,^1,6,8,61^ soon came to light and these are being increasingly emphasised/supported by emerging data. A significant amount research has now been published on the use of antibody fragments to address both fundamental and practical issues encountered during the use of whole immunoglobulins. In addition to being less immunogenic, the small size of antibody fragments allows for higher loading capacities and superior orientation of targeting ligands, leading to overall improvements in efficacy (Fig. 3).

In view of the above advantages, it is anticipated that the use of antibody fragments as directing ligands for nanoparticle targeting will increase significantly over the next few years.^9^ Whilst several excellent reviews have been written on the use of targeted nanoparticles in biomedicine, with a few focusing on the subject of antibodies as targeting ligands,^8,9^ very few specifically highlight and accurately detail work on nanoparticle–antibody fragment conjugates. This short review aims to introduce the area, with particular emphasis on recent developments in the generation and application of nanoparticle–antibody fragment conjugates for biomedical uses.

## 3. Generating nanoparticle–antibody fragment complexes

During the design of nanoparticle–antibody fragment complexes important consideration must be given to the method by which the two entities are attached. The antibody fragment needs to be conjugated to the nanoparticle in a way that causes minimal perturbation to the shape, size, and functionality of both the nanoparticle and the antibody fragment itself. Additionally, the linker between the two should be stable, biocompatible, non-toxic, and facile to install. Fortunately, a great deal of work on the installation of functional chemical moieties on both nanoparticles and antibody fragments has been carried out. Moreover, attempts to utilise these chemistries to functionalise nanoparticles with antibody fragments have been largely successful, as will be discussed in more detail below.

### 3.1 Modification of antibody fragments

**Fig. 4:**
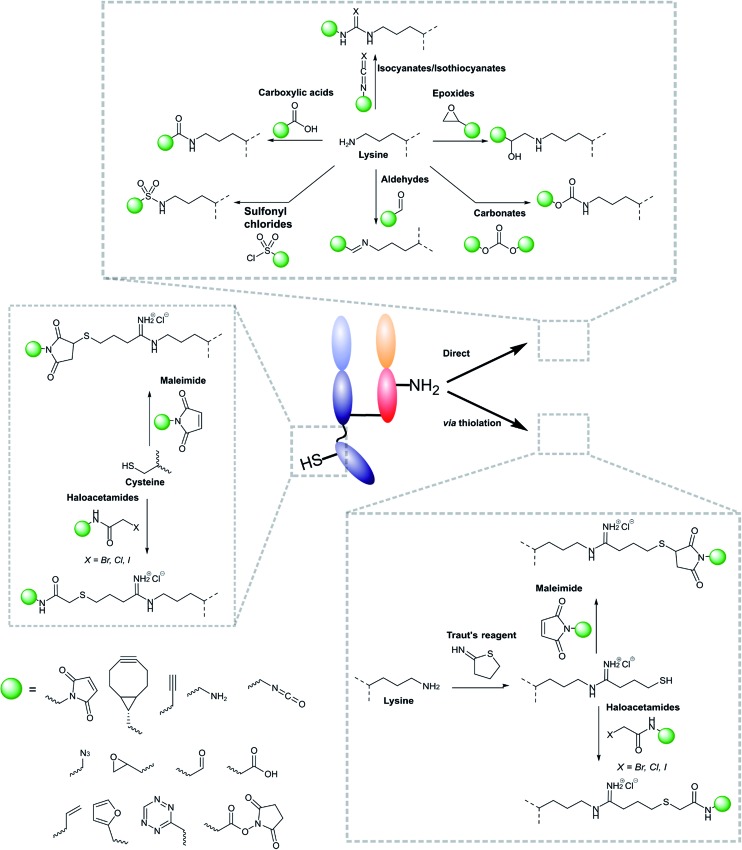
Schematic representations of common ways in which antibody fragments are modified.

Modifications of antibody fragments largely centre on exploiting the innate chemical reactivity of the natural amino acids on the backbone of each protein. The amino acids most commonly used as sites for modification include lysine, cysteine, and glutamic/aspartic acid, as they can be functionalised using well-established chemistries. Initially, lysine was a popular target for modification as it could be readily conjugated, however, the high abundance of this amino acid on the surface of many proteins means that it is hard to control conjugation, resulting in random functionalisation and a heterogeneous mixture of antibody fragment products post-conjugation. More recently, site-selective methods which exploit the natural structure of antibody fragments, such as the hinge thiols of Fab′ fragments, or utilise amino acids incorporated through site-directed mutagenesis, have been successfully employed; this has resulted in far more homogeneous and better characterised conjugates. Antibody modification (including antibody fragments) has maintained a healthy research focus for several decades now, largely due to the rapid development of the antibody–drug conjugate field. This has resulted in a rich toolbox of chemical reactions which enable facile, site-selective modification whilst avoiding negative effects on the function of the protein. Several excellent reviews have been written on this subject, so it will not be covered in depth here.^62–65^ However, Fig. 4 highlights some of the most common methods employed for functionalising antibody fragments for subsequent attachment to nanoparticles.

### 3.2 Modification of nanoparticle surfaces

**Fig. 5:**
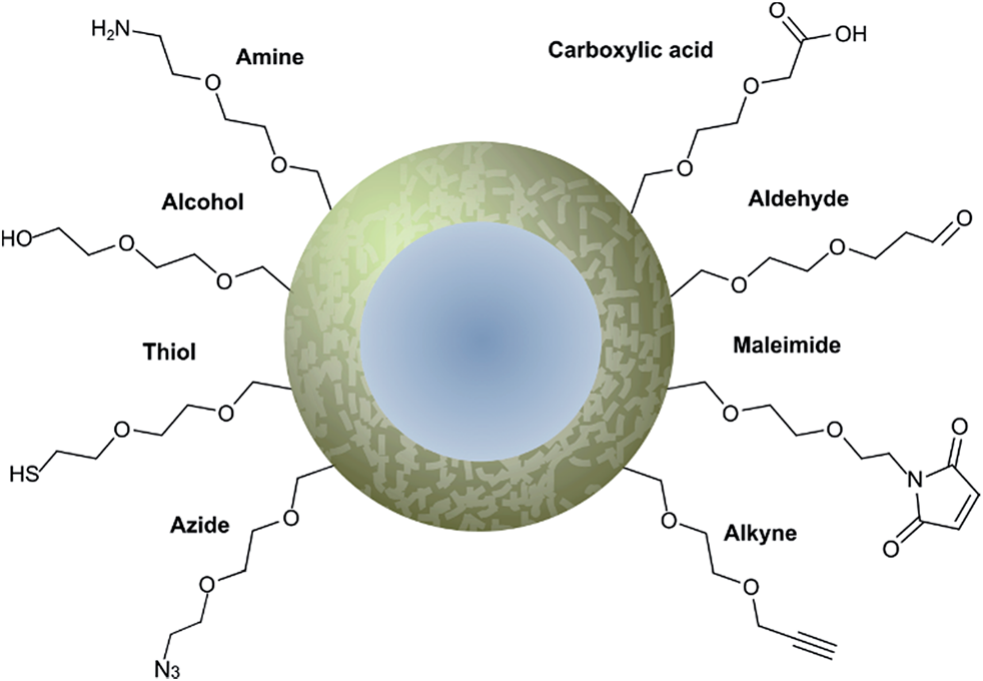
Graphical representation of common functional ligands attached to the surface of a nanoparticle.

Nanoparticle surface modification techniques can be broadly separated in two main categories: (i) covalent and (ii) non-covalent. Covalent modifications involve the incorporation of a chemical functional group that can subsequently attach covalently to a targeting ligand. In contrast, non-covalent technologies involve the incorporation of a functionality that can interact either (i) intermolecularly or (ii) by physisorption with a ligand. For decorating nanoparticles with antibodies, covalent methods are preferred as they provide greater *in vivo* stability.^8^ Moreover, covalent methods also allow for greater control over the position and orientation of the attached antibody fragment, especially when combined with a site-selectively modified antibody fragment itself. Methods for incorporating an assortment of functional groups onto the surfaces of various nanoparticles have been reported, including amines, carboxylic acids, alcohols, thiols, azides, alkynes, aldehydes, and maleimides. Subsequent modification of these groups can further expand the reactivity profile of the nanoparticle, leading to a large selection of functional handles which can be paired with complimentary groups on the desired antibody ligand (Fig. 5). Several reviews have been written on the incorporation and utilisation of chemical functionality on nanoparticles,^4,8,18,66,67^ including a comprehensive overview by Sapsford *et al.* ^68^ Despite these advances, non-specific interactions of antibody ligands with nanoparticle surfaces remains an issue, and methods for distinguishing specific interactions from non-specific interactions are lacking. These issues can be particularly problematic when site-specific or oriented conjugation of an antibody fragment is desired.

## 4. Nanoparticle–antibody fragment conjugates in biomedicine

### 4.1 As therapeutic agents

The ability to safely encapsulate a cocktail of toxic chemicals and deliver them selectively remains a long standing goal for medicine. To this end nanoparticle–antibody conjugates have shown great potential and indeed several promising candidates have entered clinical trials (Table 2). Interestingly, the majority of these candidates utilise antibody fragments as the targeting ligand, highlighting a preference over full-length antibodies for therapeutic applications. This preference is indicative of the advantages provided by the use of smaller, less immunogenic antibody-derived targeting ligands. However, it is important to note that in the cases exemplified in Table 2, side-by-side comparisons to whole immunoglobulins were not made, or at least the data was not published.

**Table 2:** A list of nanoparticle–antibody conjugates currently undergoing clinical trials. Adapted from tables previously published by Van der Meel *et al.* ^73^ and Goodall *et al.* ^11^ For details on individual therapeutics see references contained within these reviews

Name	NP type	Target	Ligand	Bioactive compound	Indication	Phase
SGT-53	Lipid	Transferrin receptor	Anti-transferrin receptor ScFv	p53 DNA	Solid tumours	Ib
SGT-94	Lipid	Transferrin receptor	Anti-transferrin receptor ScFv	RB94 DNA	Solid tumours	I
C225-ILS-Dox	Lipid	EGFR	Cetuximab Fab	Doxorubicin	Solid tumours	I
Erbitux-EDVs_pac_	Bacterially derived mini-cell	EGFR	Bispecific monoclonal antibody (mAb)	Paclitaxel	Solid tumours	II
MM-302	Lipid	HER2	Anti-HER ScFv	Doxorubicin	Breast cancer	I
Lipovaxin-MM	Lipid	Dendritic cell CD209	dAb	Melanoma antigens + IFNγ	Melanoma vaccine	I
MCC-465	Lipid	Uncharacterised (GAH)	Anti-GAH F(ab′)_2_	Doxorubicin	Metastatic stomach cancer	I
Anti-EGFR ILs-Dox	Lipid	EGFR	Cetuximab Fab	Doxorubicin	Solid tumours	I

Nonetheless, a lack of clarity regarding the advantages and disadvantages of whole mAb compared with antibody fragments for therapeutic purposes was, at least to some extent, addressed by Cheng and Allen.^69^ During the design of liposomes which could selectively target B-cell malignancies with encapsulated doxorubicin (Stealth® immunoliposomes, SIL), they compared the *in vivo* effectiveness of doxorubicin bearing liposomes targeted with HD-37 mAb, HD-37-Fab′ and a HD-37-ScFv against the B-cell antigen CD19.^69^ The targeting ligands were attached to the protein using maleimide–thiol conjugation techniques, natively in the case of the Fab′ and ScFv and *via* lysine thiolation in the case of the whole antibody. *In vitro* binding assays revealed no significant difference in CD19 binding between HD-37-mAb and HD-37-ScFv targeted liposomes, however, a steep improvement in binding was observed for HD-37-Fab′. Interestingly the HD-37-ScFv targeted liposome proved the most selective for CD19^+^ over CD19^–^ cells with the mAb being the worst performer over both studies. Drastic differences were also noticed *in vivo*, with the HD-37-mAb targeted liposome being rapidly cleared (0.41 mL h^–1^) due to Fc-mediated uptake into the liver and spleen in comparison to the fragment conjugates (0.10 mL h^–1^ for the Fab and 0.12 mL h^–1^ for the ScFv). Of the fragment-decorated liposomes HD-37-ScFv cleared slightly quicker, possibly due to His-tag/c-myc tag mediated uptake into the liver. The culmination of these effects is an improved mean survival rate of mice treated with HD-37-Fab′ targeted doxorubicin liposomes when compared to HD-37-mAb and HD-37-ScFv targeted doxorubicin liposomes. Although the presence of the His and c-myc tags caveat the results of the HD-37 ScFv targeted liposome due to increased clearance rates, this work clearly demonstrated the differences between using full mAb and antibody fragments as targeting ligands for nanoparticles. It also provided early evidence for advantages in using smaller fragments that do not contain the Fc region. These results corroborated previous work by Allen which showed that a Fab′ conjugated liposome outperformed a full mAb conjugated liposome due to increased circulation time.^70^

#### 4.1.1 Targeted delivery of small molecule drugs

In addition to the clinical examples mentioned above, a plethora of preclinical nanoparticle–antibody fragment conjugates exist for the targeted delivery of cytotoxic payloads.^9,71^ Manjappa *et al.* used an anti-neuropilin (NRP) Fab′ targeted liposome containing docetaxel to simultaneously target both solid tumours and the surrounding microvasculature.^72^ The anti-NRP Fab′ was conjugated to the liposome *via* surface PEG-maleimide groups, resulting in a site-specific thioether bridge. This allowed the targeting fragments to be arranged in a desirable orientation, an approach that is not possible with a full antibody. By taking this approach the group obtained promising results, with the targeted liposome showing the greatest degree of suppression on both tumour volume and microvessel density when compared to controls.

Whilst the majority of nanoparticle–antibody fragment drug delivery systems utilise lipid-based nanoparticles (Table 2), the last few years have seen an increased exploration of non-liposomal nanoparticle–antibody conjugates for cytotoxic drug delivery. Work by Ahn *et al.* showed that anti-tissue factor (TF) Fab′ targeted polymeric micelles loaded with dichloro(1,2-diaminocyclohexane)platinum(ii) displayed greater selectivity for the cellular target, increased internalisation rate, and afforded significant retardation of tumour growth when compared to non-targeted polymeric micelles or free drug alone.^74^ By utilising a selective maleimide–thiol reaction to attach their Fab′ ligand, the group were able to exert delicate control over the conjugation and introduce a single Fab′ per micelle. This allowed for the installation of targeting capabilities whilst causing minimal perturbation to the nanoparticle properties, an advantage for moving forward into the clinic.

Further to this, Xiangbao *et al.* successfully used an anti-VEGFR ScFv targeted polyethylene glycol–polylactic acid (PEG–PLA) polymersome containing As_2_O_3_ as the cytotoxic payload.^75^ Despite the use of suboptimal non-specific lysine–NHS ester conjugation techniques to attach the ScFv ligand, their approach yielded improved selectivity and decreased tumour volume, resulting in far greater mean survival rates when compared to the non-targeted nanoparticles and free drug controls. It is expected that controlled orientation of the ScFvs would yield even better results.

Proof of principle research by Quarta *et al.* has demonstrated the tumour targeting capability of iron oxide nanoparticles conjugated to anti-folate receptor antibody (AFRA) Fab fragments.^76^ The group chose the Fab fragment over the full antibody in order to minimise any increase in the diameter of the resulting conjugate and thus increase internalisation rate and stability. The ARFA Fab had been previously expressed to contain a hinge region with a single glutathione protected cysteine residue that could be used to conjugate to the maleimide coated nanoparticle after reductive deprotection. Interestingly, the group employed TCEP for the deprotection, a reducing agent known to cleave the interchain heavy-light disulfide bond of the Fab fragment. This would enable cysteine residues on both chains to react independently with the nanoparticle, potentially decreasing the control offered through the specific introduction of the hinge cysteine, although this it is appreciated that all liberated thiols are distal from the binding site. Whilst no cytotoxic compounds were delivered in this preliminary study, the group did demonstrate excellent *in vivo* stability, along with dramatically increased selectivity for αFR-expressing tumours when compared to non-targeted controls. Thus, whilst this approach is still in its relative infancy, it shows promise as a way of utilising inorganic iron oxide nanoparticles to deliver cytotoxic payloads for the treatment of ovarian cancer.

Other early stage research has explored the use of bispecific ScFv and SdAb targeted liposomes, and have demonstrated a clear advantage in the use of both bispecific ScFv and SdAb fragments as targeting ligands for liposomal nanoparticles.^77,78^

#### 4.1.2 Targeted gene therapy

Gene therapy relies on the selective delivery of nucleic acids to the cytoplasm or nucleus of a target cell. The delivered gene is then able to replicate within the cell and elicit its desired therapeutic effect. Whilst the majority of therapeutic nanomedicine is focused on the delivery of cytotoxic drugs, increasing effort is being spent on developing nanoparticle–antibody conjugates for targeted gene therapy.^4,79,80^ Indeed, two of the eight nanoparticle–antibody conjugates currently in clinical trials utilise specific DNA strands as their payload (SGT-53 and SGT-94, Table 1). Nanoparticle-based gene delivery was partially covered by Zhang *et al.* ^79^ and Li *et al.*,^81^ however with little focus on the details of the antibody-directed approaches, as will be discussed here.

Recently, Katakowski *et al.* showed that liposomes containing small interfering RNA (siRNA) could be targeted at dendritic cells using anti-DEC205 ScFv fragments, with *in vivo* results demonstrating improved gene silencing.^82^ Their targeting ScFv was conjugated to the nanoparticle *via* a C-terminal cysteine introduced using site-directed mutagenesis, allowing conjugation to occur distal to the binding region so as to minimise any deleterious effects on binding. The authors note that in unpublished preliminary data they were unable to utilise full anti-DEC205 antibody for the same purpose, and highlight the risks of proceeding to the clinic with full mAb targeted nanoparticles.

In addition to this, early *in vitro* work by Okamoto *et al.* suggests siRNA containing liposomes targeted to heparin-binding epidermal growth factor (HB-EGF) using anti-HB-EGF Fab′ fragments could provide effective treatment for breast cancer.^83^ Similarly, Laroui *et al.* demonstrated effective treatment of colitis through the delivery of TNF-α siRNA encapsulated within F4/80 Fab′ targeted PEG–PLA polymersomes.^84^ The group found that Fab′ targeted TNF-α siRNA containing nanoparticles granted a greater reduction in all symptoms of colonic inflammation when compared to the non-targeted controls. In both studies the Fab′ fragment was site-specifically conjugated to the nanoparticle *via* the hinge region using maleimide–thiol chemistry, highlighting the emerging prevalence of this approach for conjugating antibody fragments to nanoparticles.

Further to the above examples, work carried out at Sun Yat-sen University has pioneered the use of ScFv targeted superparamagnetic iron oxide nanoparticles (SPIONS) as MRI visible siRNA delivery vectors.^85,86^ One study demonstrated the applicability of this approach towards the treatment of neuroblastoma tumours, with *in vivo* data suggesting significant gene silencing and subsequent tumour suppression.^85^ Early data suggests a similar approach could be utilised for the treatment and imaging of gastric cancer.^86^ These studies show that delivery of nucleotides is not limited to organic nanoparticles, and that the innate physical properties of inorganic nanoparticles can grant significant benefits.

#### 4.1.3 Magnetic field therapy

**Fig. 6:**
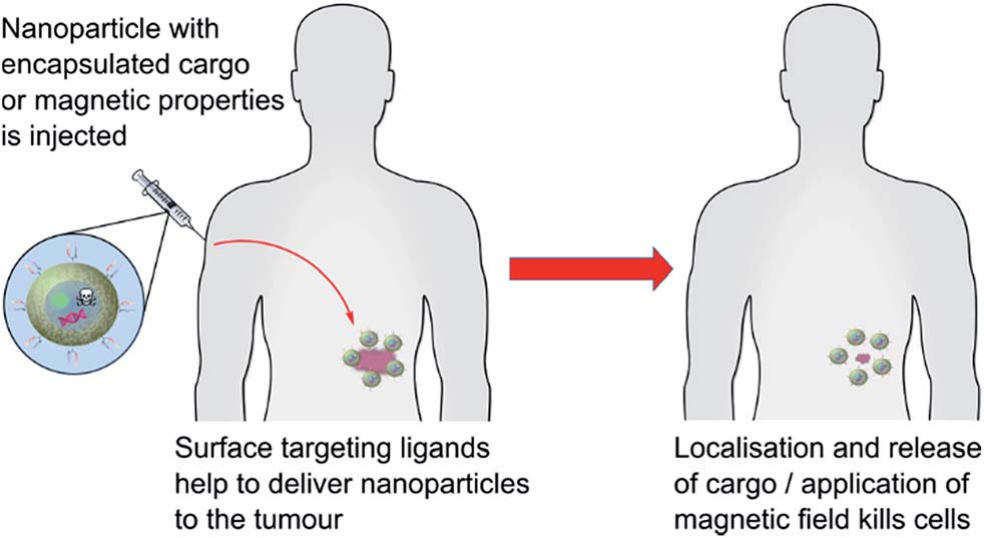
A graphical representation of actively targeted nanoparticle therapeutics.

Within the confines of targeted nanomedicine, magnetic field therapy relies on the localised induction of heat to a cell through the use of targeted nanoparticles which respond thermally to the application of an alternating magnetic field. Utilisation of targeting ligands, such as antibodies, has enabled nanoparticles to localise at a tumour site, and upon application of an alternating magnetic field cause heating which destroys the proximal diseased cells (Fig. 6).^87^ Whilst liposomal nanoparticles are preferred for drug and gene delivery, the inherent superparamagnetic properties of iron oxide nanoparticles (SPIONs) have led to their predominant usage in this area. The idea of using antibodies to direct magnetic nanoparticles was explored extensively by Gerald and Sally DeNardo in the mid- to late-2000s,^88–91^ and significant progress has been made ever since. Whilst most of this development has focused on the use of full antibodies as targeting ligands, with optimisation more focused on the nanoparticle side,^92–95^ some preliminary work has demonstrated advantages in the use of antibody fragments in this context. For example, early work by Shinkai *et al.* showed effective use of a Fab′ of antibody G250 to deliver a magnetoliposome to MN-antigen presenting cells.^96^ Application of an alternating magnetic field to this complex resulted in tumour suppression and almost doubled the mean survival rates of mice when compared to negative controls. An excellent paper by Cui *et al.* also exemplified the “theranostic” utility of targeted SPIONs *via* the application of an anti-prostate specific antigen (PSA) ScFv-decorated fluorescent magnetic nanoparticle.^97^ By combining the fluorescent payload with the superparamagnetic properties of the iron oxide nanoparticle, the group were able to track delivery *in vivo* using fluorescence and magnetic resonance imaging, as well as initiate cell death through the application of an alternating magnetic field. This approach afforded a substantial increase in lifespan in diseased mice when compared to controls. It is worth noting that the ScFv was conjugated to the nanoparticle *via* non-specific reactions between nucleophilic amino acid residues and surface-bound glutaraldehyde linkers, leading to uncontrolled surface loadings and orientation. Thus, it is possible that these results could be improved through the use of a more controlled conjugation method.

Towards the end of their studies into magnetic field therapy, Gerald and Sally DeNardo published work in which the full mAb was abandoned in favour of a di-ScFv ligand, which was attached in a highly oriented fashion *via* a carefully introduced cysteine residue.^98^ Whilst the SPION-ScFv showed greatly increased accumulation at the tumour site *in vivo*, efficacy of the hyperthermic properties of the nanoparticle was not explored. Similar work by Yang *et al.* showed that magnetic iron oxide nanoparticles can be selectively targeted towards the EGFR using an anti-EGFR ScFv ligand, showing promise as a treatment for various EGFR presenting cancers.^99^

The results discussed above clearly demonstrate the advanced capabilities of nanoparticle–antibody fragment conjugates for chemotherapy. It is anticipated that the trend of using antibody fragments could also provide benefits in other areas of nanomedicine, *e.g.* targeted immunotherapy through the activation of cell receptors such as Death Receptor 5 (DR-5).^100,101^

### 4.2 As imaging agents

Conceptually, targeted nanoparticles provide a myriad of benefits for *in vivo* imaging of cellular targets. The generous loading capacity of most particles enables the site-selective delivery of large quantities of imaging agent, increasing signal-to-noise ratio, and/or the nanoparticle surface itself can often be tailored to provide intrinsic imaging functionality, as is the case with SPIONs, gold nanoparticles or quantum dots. Early work in the use of antibody–decorated nanoparticles for imaging applications encountered problems due to specific accumulation, with the limiting step found to be extravasation of the nanoparticles from the vasculature, rather than cell binding.^1,4,7,41,93,102^ Whilst this is also a problem for therapeutic nanoparticles, it is more apparent for imaging applications where the utility is highly dependent on achieving high resolution between the target site and the background. In an attempt to tackle this problem, recent work has focused on the use of smaller antibody fragments as targeting ligands. By utilising smaller antibody fragments, which do not contain the Fc region, overall circulation times and subsequent tumour accumulation rates can be increased greatly.

#### 4.2.1 Targeted optical imaging agents

Antibody fragment–decorated nanoparticles can be employed as optical imaging agents either by: (i) encapsulation of certain small molecules; (ii) incorporation of highly fluorescent compounds onto the nanoparticle or targeting antibody; or (iii) the use of innately fluorescent materials to construct the nanoparticle itself. An excellent example of the former approach is shown in a study by Fiandra *et al.* which compared the use of antibody fragments to the parent full antibody for imaging HER2 positive tumours.^103^ Iron oxide nanoparticles modified with a fluorescent dye were targeted towards HER2 cells using full trastuzumab, trastuzumab half antibody (consisting of a single heavy chain and a single light chain), or a trastuzumab derived ScFv. *Ex vivo* results suggested a significant improvement in tumour accumulation for the half antibody and the ScFv–decorated nanoparticles when compared to the full antibody targeted nanoparticles. Importantly, each of the targeted nanoparticles showed at least a 30-fold increase in fluorescence when compared to the non-targeted control. It should be noted that different conjugation techniques were used for the different ligands; both the half antibody and the ScFv were attached *via* thiol selective covalent disulfide formation, whereas the full antibody was attached *via* stable non-covalent protein A affinity interactions. These results further demonstrate the benefits of actively targeted nanoparticles for imaging tumours, and the importance of ligand choice.

Another excellent example is provided by the work of Rüger *et al.* who used a self-quenching near-infrared dye incorporated inside a ScFv–decorated liposome to image fibroblast activation protein alpha (FAP) expressing cells. Application of a self-quenching fluorophore ensured significant fluorescence was only observed after intra-cellular degradation of the liposome post-FAP cell binding. This approach led to a significant increase in the signal-to-noise ratio of the ScFv–decorated liposomes when compared to the non-targeted controls *in vivo*. The authors specify their decision to utilise an ScFv rather than a whole mAb was driven by potential immunogenic concerns.^104^

Exploiting inherently fluorescent nanoparticles such as gold nanoparticles or quantum dots is more widely utilised, likely due to their relatively large extinction coefficients and resistance to photobleaching. Several excellent examples of antibody fragment-decorated approaches exist. As way of an example, Xu *et al.* showed that anti-GRP78 ScFv-conjugated quantum dots can be tracked *in vivo* using fluorescence imaging.^105^ A similar approach was used by Balalaeva *et al.* to image breast cancer *in vivo*.^106^ Other groups are currently exploring the use of an anti-CEA sdAb conjugated quantum dot for imaging CEA expressing cancer cells, with initial results showing great promise.^107–109^ Use of an sdAb allowed for highly orientated attachment of the targeting ligand through an engineered cysteine residue, greatly increasing avidity. The superiority of their sdAb is supported by recent results comparing the sdAb ligand with a full antibody analogue; the study demonstrated a dramatic increase in sensitivity when the smaller targeting ligand was employed.^110^ In both cases lysine residues on the targeting antibody ligands were modified with D-biotin using NHS ester chemistry, allowing the ligands to be attached to the quantum dot using the highly stable biotin–streptavidin interaction. Whilst this non-covalent approach is not ideal, it allowed the researchers to utilise the same coupling strategy for both ligands and thus gain a fairer comparison of their sdAb against the full antibody.

#### 4.2.2 Targeted nuclear imaging agents

Nanoparticles have also been employed with great success in the selective delivery of radionuclides for imaging techniques such as positron emission tomography (PET) and single photo emission computed tomography (SPECT).^111,112^ The selective delivery of radionuclides can also have a desirable therapeutic effect, allowing targeted nanoparticles loaded with radionuclides to act as successful theranostic tools.^113,114^ Whilst a multitude of examples exist in which full antibody–decorated nanoparticles have been utilised for this purpose, less work has been carried out using smaller antibody-based fragments.^115,116^ Nonetheless, there is movement towards this area and a few notable examples are outlined below.

Chen *et al.* utilised a highly functionalised mesoporous silica nanoparticle (MSN) to successfully image tumour vasculature *in vivo* using a multimodal approach which employed both PET and optical imaging techniques.^117^ To target the nanoparticles, the group attached a Fab fragment targeted against CD10, a vascular-specific marker for tumour angiogenesis, and demonstrated a significant improvement in both PET and fluorescence imaging resolution *in vivo* compared to non-targeted controls.

Work by Hoang *et al.* has utilised ^111^In-labelled block copolymer micelles conjugated to trastuzumab Fab to image HER2 positive cell lines *in vitro* using SPECT/CT.^118^ In addition to the trastuzumab Fab targeting ligand the group incorporated nuclear localisation signal (NLS) peptides onto the surface of their nanoparticle, leading to effective nuclear translocalisation after initial HER2 mediated internalisation. More recently, this approach was demonstrated *in vivo*, with significant benefits in tumour accumulation, cellular uptake, and nuclear uptake being reported, when compared to non-targeted controls. Tumour uptake studies indicate the nanoparticles functionalised with both extra-cellular (trastuzumab Fab) and intra-cellular (NLS peptides) targeting ligands outperformed the nanoparticles targeted using trastuzumab Fab alone, indicating post-internalisation nuclear translocation could be beneficial.^119^

#### 4.2.3 Targeted MRI agents

A great deal of effort has been put into exploring the use of nanoparticles as contrast agents for magnetic resonance imaging (MRI). Although the majority of this work has focused on the use of innately magnetic nanoparticles such as SPIONS and carbon nanotubes, organic nanoparticles have also found some use due to their ability to safely encapsulate existing MRI contrast agents.^120^ Actively targeted approaches have gained popularity in recent years, with antibody-derived ligands showing particular promise.^121^ An early example of the use of an antibody fragment to target a magnetic nanoparticle was provided by Yang *et al.*, who used an anti-EGFR ScFv to selectively deliver iron oxide nanoparticles to EGFR-expressing cancer cells.^99^ *In vivo* results showed significant improvement in MRI contrast when ScFv targeted iron oxide nanoparticles were compared to non-targeted controls. The group utilised non-selective lysine–NHS ester chemistry to attach the ScFv, so it is likely that further improvements could be achieved through the use of a more controlled conjugation strategy. Vigor *et al.* utilised a similar approach to target their SPIONs towards CEA expressing cells.^122^ By attaching an anti-CEA ScFv fragment to the surface of their SPION the group were able to demonstrate excellent target specificity and MRI contrast *in vitro* when compared to non-targeted controls. More recently, Alric *et al.* showed that an anti-HER2 ScFv could be effectively employed to traffic PEG coated SPIONS to HER2 expressing cells.^123^ These targeted SPIONS maintained binding affinity and demonstrated increased cellular uptake when compared to non-targeted controls. It should be noted that the authors explicitly employ a small ScFv and site-selective maleimide–thiol coupling to achieve optimal orientation, cause minimal perturbation to nanoparticle size, and avoid any problems associated with the employment of full antibodies.

### 4.3 As immunoassays

**Fig. 7:**
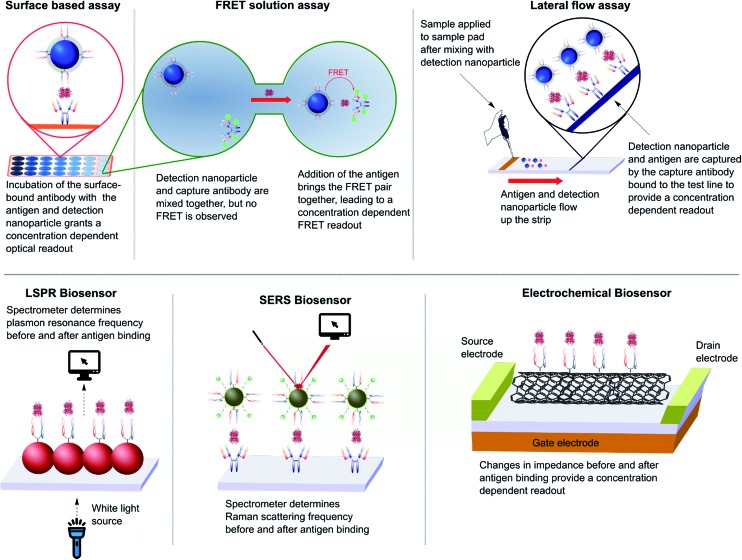
Various designs of immunoassay ranging from surface based, FRET and lateral flow assays to LSPR, SERS and electrochemical biosensing.

The impact of nanoparticles on biomedicine is perhaps most pronounced in the field of immunoassays and diagnostics. The varied optical, physical, and electrochemical properties of nanoparticles present a wide range of observable outputs which can be exploited for the detection of disease biomarkers. The *in vitro* nature of diagnostic tools eliminates the negative impact of the suboptimal *in vivo* properties found with many inorganic nanoparticles (*e.g.* toxicity, bioaccumulation), allowing their full potential to be more readily realised. To date, nanoparticle–full antibody conjugates have found use in immunoassays based on fluorescence,^124^ Förster resonance energy transfer (FRET),^125^ catalytic redox reactions,^126,127^ surface plasmon resonance (SPR),^128^ surface-enhanced Raman (SER),^128,129^ and surface electrochemistry,^130,131^ amongst many others (Fig. 7).^124,132,133^ Examples of nanoparticle–antibody fragment conjugates are less abundant; this may be as a result of the relative infancy of the field and mAb immunogenicity no longer being an issue. However, recent reports suggest that significant gains can still be obtained through a switch in focus from full antibodies to antibody fragments, some of which are described below.

#### 4.3.1 Fluorescence/FRET immunoassays

Optical immunoassays rely on colourimetric or fluorescence-based reporter molecules for the detection of the target analyte. These assays are often simple and require relatively basic equipment to interpret, an advantage for the design of point of care/point of demand (POC/POD) diagnostic devices. A simple example of this is provided by Anderson *et al.*, who utilised sdAb–QD conjugates in an immunoassay for the detection of ricin.^134^ The group exploited the fluorescence of the quantum dot as a reporter in a sandwich assay, observing limits of detection comparable with traditional fluorescent dyes. In the same study, the group showed that the same sdAb–QD conjugate could be used in a surface plasmon resonance (SPR) assay, achieving a 10-fold increase in sensitivity compared to the sdAb alone. Thus the group were able to utilise their sdAb–QD conjugate in a dual-detection capacity, exploiting both the optical and physical properties of the quantum dot. Interestingly, and in support of controlled antibody fragment orientation, the group attached the ScFv to their quantum *via* a selectively introduced His-tag, exploiting the interaction with the zinc ions on the surface of the quantum dots.

Further to the above, Wegner *et al.* have employed the FRET capabilities of QDs in their sandwich immunoassays to great effect.^135^ Their assays rely on an antigen-mediated FRET coupling between a QD conjugated reporter antibody and a terbium-labelled capture antibody. The group compared full antibody, F(ab′)_2_, and Fab fragments as targeting ligands for their QD–antibody conjugates in an immunoassay for prostate specific antigen (PSA). In each case, non-specific conjugation techniques were employed. It was found that the QD–Fab significantly outperformed the QD–full antibody, achieving a 5-fold increase in sensitivity for PSA in serum samples. The authors attributed this to a combination of decreased distance between the FRET pairs and improved orientation of the Fab on the surface of the QD. The group utilised a similar assay for the detection of EGFR in serum, achieving comparable success when employing a QD–nanobody construct as their reporter molecule.^136^ These solution based assays hold advantages over the more traditional surface based assays as they do not require immobilisation of the capture antibody onto a surface. This increases efficiency and practicality, whilst eliminating potential inaccuracies brought about by non-specific sticking of the nanoparticles to the plate.

#### 4.3.2 LSPR immunoassays

Localised surface plasmon resonance (LSPR) relies on changes occurring on the surface of a nanoparticle upon successful binding of a disease marker to a surface-immobilised targeting ligand. In the case of LSPR immunoassays, binding of the antigen to the antibody ligand results in small changes in the dielectric field surrounding the conjugated magnetic nanoparticle. This changes the frequency of the surface plasmon produced during interaction of the particle with electromagnetic radiation, a phenomenon which can be measured with great accuracy.^133,137^ Byun *et al.* showed that LSPR could be used to detect C-reactive protein (CRP), a protein used as a biomarker for inflammatory diseases.^138^ The group utilised a gold nanorod conjugated to an ScFv *via* a selective cysteine residue and were able to detect CRP in serum at concentrations lower than 1 ng mL^–1^. The authors note that a conscious decision was made to use the small ScFv rather than a whole antibody as LSPR effects are more pronounced when the antigen–antibody interaction occurs closer to the surface of the nanoparticle. The smaller size of the ScFv compared to the full antibody helped to achieve this.

#### 4.3.3 SER immunoassays

SER immunoassays exploit the observed amplification of the Raman scattering profile of a system upon binding of a disease marker. Typical SER immunoassay systems involve a metallic nanoparticle which has been functionalised with both an antibody capture ligand and a sensitive Raman reporter molecule. Differences in the Raman spectra before and after binding of the antigen can be used to quantify the amount of antigen present. This technique has been shown to be highly sensitive, allowing single molecules to be detected in certain cases.^133,137,139,140^ Bishnoi *et al.* exploited this successfully to generate an immunoassay against a protein implicated in retinal damage.^141^ The group used lysine–NHS ester chemistry to conjugate the Fab fragment of their expressed antibody to the surface of a gold nanoparticle which had been pre-functionalised with the Raman reporter *p*-mercaptoaniline. Using this approach, a linear relationship between retinal lysate concentration and the Raman signal was observed, with negative controls producing only negligible effects on the signal. Similarly, Qian *et al.* were able to successfully exploit SERS to detect the presence of EGFR on the surface of human cells *in vitro* using a gold nanoparticle–ScFv conjugate.^142^ Whist it is appreciated that the work performed was not strictly an immunoassay, the results suggest that that an EGFR immunoassay based on SERS could be readily developed.

#### 4.3.4 Electrochemical immunoassays

Electrochemical immunoassays utilise the electronic or electrochemical properties of inorganic nanoparticles to determine antigen binding to surface-bound antibody/antibody fragment ligands. In a typical set up a conductive nanoparticle, such as a carbon nanotube, is conjugated to a capture antibody ligand. Binding of the antigen to the antibody causes minute changes in the electrical environment on the surface of the nanotube, altering the electrical conductance and thus producing a quantifiable signal. Electrochemical techniques have been found to be highly sensitive, robust, and easy to use. Through combination with microfluidic cells, electrochemical immunoassays have been fabricated into full integrated immunosensors for point of care applications.^130,133,143^

Lo *et al.* employed the use of an electrochemical immunoassay for the detection of CEA. By immobilising an anti-CEA ScFv onto the surface of nickel coated carbon nanotubes the group were able to demonstrate a quantifiable difference in electrical conductivity before and after incubation with the disease marker.^144^ This approach provided a detection limit of 10 ng mL^–1^, a 10-fold increase in sensitivity compared to a near identical study where a full antibody against CEA was employed.^145^ The authors attribute this increased sensitivity to the smaller size of the ScFv and its orientation on the nanoparticle through a selective interaction between the nickel coating and the His tag on the ScFv. When this selectivity was removed through the introduction of multiple chelating sites, a nullification of the activity was observed, thus demonstrating the importance of oriented immobilisation. More recently, Lerner *et al.* utilised a carbon nanotube to design an immunoassay for the detection of osteopontin (OPN), a disease marker for prostate cancer.^146^ The group attached an anti-OPN ScFv to a carbon nanotube and were able to detect OPN in serum samples at concentrations as low as 1 pg mL^–1^, a detection limit three orders of magnitude lower than commercial ELISA assays against the same marker.

## 5. Conclusions and future outlook

Whilst traditional nanoparticle–full antibody conjugates have proven to be effective tools for both therapeutic and research purposes, limitations resulting from the use of whole immunoglobulins briefly plateaued progress in the area. However, a switch in focus to antibody-based fragments, both natural and engineered, is leading to a positive step-shift in progress. It is clear from the evidence presented in this review that antibody fragments have great potential as targeting ligands for nanoparticle based therapeutics, diagnostics and bioassays, with the resulting constructs demonstrating greater selectivity, superior antigen binding, and more favourable pharmacokinetic properties.

It seems we are now at a stage where we are fine-tuning how the antibody fragment is specifically connected to the nanoparticle; as exemplified, the choice of conjugation technique plays an important role in the properties of the resulting nanoparticle–antibody fragment conjugate with more controlled chemistries consistently providing superior results. The marriage of site-selective conjugation strategies with the unique properties and smaller size of antibody fragments allows for the installation of highly oriented targeting ligands, a clear advantage for selectivity, *in vivo* tolerance and binding affinity. We predict that the future in this field will see a continuation in the trend towards antibody fragment based targeting ligands being installed *via* increasingly selective and controlled chemistries; potentially providing access to hitherto unexplored applications for antibody targeted nanoparticles.
